# Cardiovascular Disease Risk amongst African Black Patients with Rheumatoid Arthritis: The Need for Population Specific Stratification

**DOI:** 10.1155/2014/826095

**Published:** 2014-07-23

**Authors:** Ahmed Solomon, Linda Tsang, Angela J. Woodiwiss, Aletta M. E. Millen, Gavin R. Norton, Patrick H. Dessein

**Affiliations:** ^1^Department of Rheumatology, Charlotte Maxeke Johannesburg Academic Hospital, Faculty of Health Sciences, University of the Witwatersrand, Parktown 2193, Johannesburg, South Africa; ^2^Cardiovascular Pathophysiology and Genomics Research Unit, School of Physiology, Faculty of Health Sciences, University of the Witwatersrand, P.O. Box 1012, Melville 2109, Johannesburg, South Africa

## Abstract

Rheumatoid arthritis (RA) enhances the risk of cardiovascular disease to a similar extent as diabetes. Whereas atherogenesis remains poorly elucidated in RA, traditional and nontraditional risk factors associate similarly and additively with CVD in RA. Current recommendations on CVD risk stratification reportedly have important limitations. Further, reported data on CVD and its risk factors derive mostly from data obtained in the developed world. An earlier epidemiological health transition is intrinsic to persons living in rural areas and those undergoing urbanization. It is therefore conceivable that optimal CVD risk stratification differs amongst patients with RA from developing populations compared to those from developed populations. Herein, we briefly describe current CVD and its risk factor profiles in the African black population at large. Against this background, we review reported data on CVD risk and its potential stratification amongst African black compared to white patients with RA. Routinely assessed traditional and nontraditional CVD risk factors were consistently and independently related to atherosclerosis in African white but not black patients with RA. Circulating concentrations of novel CVD risk biomarkers including interleukin-6 and interleukin-5 adipokines were mostly similarly associated with both endothelial activation and atherosclerosis amongst African black and white RA patients.

## 1. Introduction

Rheumatoid arthritis (RA) is a chronic inflammatory and destructive joint disease that augments the risk of atherosclerotic cardiovascular disease (CVD) to a similar extent as diabetes [1–4]. Recent meta-analyses have documented a 2-fold increased risk of CVD events and a standardized cardiovascular mortality rate of 50% in RA [5, 6]. Patients with RA were also shown to experience a markedly enhanced cumulative incidence of silent myocardial infarction (MI) and sudden death as well as heart failure; the latter is strikingly often with preserved ejection fraction [1]. Thus, the presenting CVD features in RA differ from those in the general population. Further, death after an acute coronary syndrome is increased in RA [7]. What drives the enhanced risk for CVD in patients with RA?

Atherogenesis in RA remains poorly understood. Traditional and nontraditional risk factors associate similarly and additively with atherosclerosis and CVD events in RA patients [8–11]. Genetic factors contribute to the enhanced CVD risk in RA [12, 13]. However, amongst nonconventional risk factors, it is high-grade inflammation that is mostly implicated in increased atherogenesis in RA [14, 15]. Indeed, patients with RA experience high-grade inflammation driven by augmented cytokine production, which associates with metabolic risk including insulin resistance and reduced HDL cholesterol concentrations [16–18]. A recent meta-analysis confirmed the impact of RA on metabolic risk [19]. Indeed, HDL concentrations were found to be reduced and the prevalence of diabetes increased in RA. Further, inflammatory molecules can also directly impair endothelial function [11, 20, 21].

Over the recent past, an impressively increasing number of investigators have reported on potential determinants of increased CVD in RA. However, over the past decades, in contrast to the substantial improvements in life expectancy in the non-RA population, which are further largely driven by reductions in CVD events in the developed world, the mortality of RA has remained remarkably constant thereby translating into a widening mortality gap between RA patients and the general population [3]. Congruent with this observation, currently recommended strategies on cardiovascular risk stratification in patients with RA were shown to have important limitations [22, 23]. A multicenter undertaken by “a transatlantic cardiovascular risk calculator for rheumatoid arthritis” (ATACC-RA) consortium recently successfully produced an RA specific cardiovascular risk calculator [24].

However, a further concern in the present context is that most information on CVD in both non-RA and RA subjects originates in developed countries that are largely inhabited by white populations whereas 80% of the CVD risk burden now arises in middle income and low income countries [25]. In this regard, at least in part due to previous colonialism and the subsequent related apartheid system that was only officially terminated in 1994, African black persons currently represent a mostly developing population. The increase in incident CVD in poorer populations is attributable to the epidemiological health transition [26]; the stages and characteristics of which are presented in Table 1. Presently, most sub-Saharan black South Africans are reportedly in stages 1 and 2 of this transition [27]. Being in epidemiological transition translates into sustaining different cardiovascular risk factor profiles and, consequently, altered CVD presentations [26]. It is therefore conceivable that data on atherosclerotic CVD and its stratification obtained in patients with RA from developed populations cannot be directly extrapolated to those that belong to developing populations.

Herein, we briefly describe current CVD and its risk factor profiles in the African black population at large. Against this background, we then review reported data on CVD risk and its potential stratification amongst African black compared to white patients with RA. Finally, we suggest future research perspectives in the present context.

## 2. Cardiovascular Disease and Its Risk Factor Profiles amongst the African Black Population at Large

### 2.1. Cardiovascular Disease Burden

There is an overall paucity of large scale epidemiological data on CVD and its risk factors in African black people. In the 2010 South African Medical Research Council report on causes of death, cerebrovascular disease was listed as the 5th and coronary artery disease as the 8th most common causes of death [28]. Moreover, peripheral arterial disease was identified in 29% of outpatients in a rural community in South Africa [29].

Strong evidence towards an early epidemiological transition stage currently experienced by African black persons comes from studies on CVD event types in this population. Although coronary artery disease (CAD) is reportedly distinctly uncommon, it was recently identified in 10% of black patients presenting to hospital with heart disease in a study that originated in Johannesburg, South Africa [30]. Also, whereas stroke incidence was found to remain lower, stroke occurs at a younger age and results in high and possibly larger mortality than in high-income regions [31–33]. There is further an emerging risk of ischemic as opposed to hemorrhagic stroke that relates to older age and the presence of diabetes [31, 34]. In a recent hospital-based study, that included 207 African black and 47 white stroke patients, the frequency of ischemic stroke and cerebral hemorrhage was 68% versus 77% and 27% versus 15%, respectively [31]. These differences were not significant. Heart failure mostly attributed to hypertension and idiopathic dilated cardiomyopathy is the most frequently made diagnosis amongst African black patients presenting with heart disease [30].

### 2.2. Cardiovascular Risk Factors

With regard to CVD risk factors, the low incidence of CAD has generally been attributed to low total cholesterol and high HDL cholesterol concentrations in African black persons [35]. However, recent studies reported reduced HDL cholesterol concentrations in this population, and low total cholesterol levels could be attributed to concurrent low HDL cholesterol levels [36]. Indeed, an alternative and more conceivable explanation is a more limited lifetime exposure to CVD risk factors, which is in line with the current rapid urbanization in this population [37]. In this regard, the prevalence of all major traditional CVD risk factors, except from low tobacco consumption, was found to be high and rising further in recent African black population studies [38–40]. In addition, particularly South African black women generally sustain much larger obesity rates than other groups living in the same region [41]. With regard to psychosocial stress as a potential CVD risk factor, the recent World Health Organization Mental Health Survey revealed that within the historical context of early after apartheid, anxiety and other mood disorders were relatively more prevalent and severe in South Africa than in other participating countries [42].

### 2.3. Impact of Cardiovascular Risk Factors on Cardiovascular Disease

In the INTERHEART Africa study and the associations of modifiable CVD risk factors with acute MI were similar to those in the overall INTERHEART study, with smoking, diabetes, hypertension, abdominal obesity, and dyslipidemia providing a population attributable risk of 89.2% for acute MI [25]. However, a history of hypertension revealed a higher MI risk in the African black group. In relatively large stroke studies amongst African black patients, hypertension was the most frequently implicated cause [31, 32].

Both systolic and diastolic blood pressures are further important determinants of diastolic function in this population [43]. Importantly, the potential influence of excess adiposity on stroke risk was not reported and the cause remained unidentified in 43% of cases. Excess adiposity is associated with hypertension as well as diastolic left ventricular function and systemic inflammation in this population [44, 45]. The risk of tobacco related CVD (as well as cancer) in urban African black persons is similar to that reported in developed populations [46]. Even mild current smoking was strongly associated with blood pressure in an African black population study [47]. Interestingly, psychosocial stress but not hypertension was associated with angiopoietin-2 and vascular endothelial growth factor-A concentrations, which are markers of angiogenesis that associate with vascular dysfunction in African black subjects [48]. Whether novel CVD risk biomarkers can improve CVD risk stratification beyond conventional CVD risk factors in this population remains however largely unknown.

## 3. The Impact of Rheumatoid Arthritis on Cardiovascular Risk Factors, Atherosclerosis, and Their Relations amongst Black Africans

The potential impact of RA on cardiovascular risk factor profiles including traditional risk factors and systemic inflammation, atherosclerosis, and their relationships was investigated in 274 African black patients of which 115 had established RA [49].

### 3.1. Cardiovascular Risk Factor Profiles

Amongst conventional risk factors, overall and abdominal adiposity as estimated by body mass index and waist-height ratio, respectively, were markedly reduced in RA. Dyslipidemia was less prevalent in RA, a finding that was explained by reduced adiposity and chloroquine use. RA patients were more often former smokers [(odds ratio (OR)) (95% confidence interval (CI)) = 2.48 (1.03–5.99)]. However, other conventional risk factors including fat distribution (waist-hip ratio), current smoking, diabetes, and hypertension prevalence as well as the number of major traditional risk factors did not differ by RA status.

With regard to nonconventional cardiovascular risk factors, circulating CRP concentrations were similar in both groups and those of IL-6 were actually reduced in RA, possibly as a result of reduced adiposity.

### 3.2. Atherosclerosis Burden

The mean ultrasound determined carotid artery intima-media thickness (cIMT) was 0.700 (0.085) and 0.701 (0.111) mm which was similar in RA and non-RA subjects in univariate and adjusted analysis.

### 3.3. Risk Factors Associated with Atherosclerosis

Clinical RA activity characteristics were consistently unrelated to systemic inflammatory markers, even in patients with moderate or high disease activity (Clinical Disease Activity Index > 10). By contrast, non-RA characteristics comprising adiposity indices, smoking and alcohol consumption status, and angiotensin converting enzyme inhibitor use were related to systemic inflammation and to a similar extent in persons with and without RA.

Amongst cardiovascular risk factors, only low density lipoprotein concentrations were weakly associated (partial *R* = 0.153 to 0.135; *P* = 0.03 to 0.06) depending on covariates included in mixed regression models with atherosclerosis in all participants and, again, RA did not impact this and all the other risk factor-atherosclerosis relationships.

Taken together, amongst black Africans from a developing population, RA can currently have an impact on individual conventional risk factors but is not associated with an increased overall increased traditional and nontraditional cardiovascular risk factor and atherosclerosis burden. The distinctly low prevalence of extra-articular manifestations in black Africans with RA points towards an inflammatory process that is mostly restricted to the joints [50]. Indeed, particularly our findings of similar CRP and lower IL-6 concentrations in RA compared to non-RA subjects as well as the consistent lack of relationships between clinical disease activity markers and the respective acute phase responses suggest that an absent IL-6 release by inflamed RA joints into the circulation can account for the unaltered risk. Is the atherosclerotic cardiovascular risk factor burden presently still more favorable in black compared to other Africans with established RA?

## 4. Cardiovascular Risk Factor Profiles amongst Black and Other Africans with RA 

### 4.1. Major Conventional Risk Factors

Potential disparities in atherosclerotic CVD risk factor profiles between 291 black and 335 (229 whites, 64 Asian, and 42 mixed ancestry) other Africans with RA were determined [51]. Compared to other Africans, black Africans smoked less frequently but had more prevalent hypertension and diabetes together with concurrent lower total as well as HDL cholesterol concentrations that resulted in unaltered atherogenic indices. These results are congruent with those on cardiovascular risk factor profiles in non-RA black persons in sub-Saharan Africa as outlined previously. More importantly in the present context, these findings translated into global scores for major conventional risk factor-mediated future atherosclerotic CVD event rates that were not reduced in black compared to other African RA patients.

### 4.2. Metabolic Syndrome and Its Components

The metabolic syndrome (MetS) reportedly predicts incident diabetes and atherosclerotic CVD, and its presence calls for lifestyle intervention. In this study, the MetS blood pressure and HDL criteria were more prevalent whereas the respective triglyceride criterion was less frequent amongst African black compared to other African patients with RA. In developed populations, increased triglyceride and decreased HDL cholesterol production typically concur [36]. However, low triglyceride concentrations despite the presence of reduced HDL levels were also previously reported in African black non-RA subjects [36]. Importantly, in the present context also, the overall metabolic risk burden as estimated by MetS prevalence and the number of MetS criteria was similar in African black compared with other African patients with RA.

### 4.3. Nonconventional Risk Factors

Black ethnicity did not independently associate with nonconventional cardiovascular risk factors including rheumatoid factor status, markers of inflammation, and brachial pulse pressures. Mixed-ancestry Africans without RA reportedly still sustain a lower risk for ischemic heart disease than white and Asian Africans [25]. When we excluded Africans of mixed ancestry from our analysis, the findings were unaltered.

Taken together, overall conventional and nonconventional CVD risk burdens and arterial stiffness were similar in black compared to other African patients with RA. This indicates that CVD risk should be assessed and managed irrespective of ethnic origin and epidemiological transition stage in RA. However, amongst Africans, is the atherosclerosis burden and the impact of cardiovascular risk factors on atherosclerosis, as large in black compared to white patients with RA?

## 5. The Atherosclerosis Burden and Its Associations with Conventional Risk Factors and Inflammation in Black and White Africans with RA

**Figure 1:**
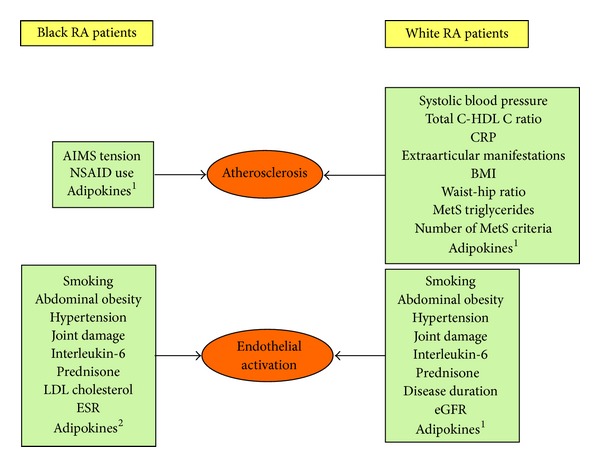
Cardiovascular risk factors that were associated with atherosclerosis and endothelial activation in African black and white patients with RA. ^1^Adiponectin, leptin, resistin, retinol binding protein-1, and chemerin. ^2^Leptin, resistin, retinol binding protein-1, and chemerin.

The carotid atherosclerosis burden and its relationship with major conventional and nonconventional cardiovascular risk factors between Africans with RA were compared between 121 black and 122 white Africans with RA [52]. The risk factors that were associated with atherosclerosis in African black and white patients with RA are shown in Figure 1.

### 5.1. Atherosclerosis Burden

The mean ± SD cIMT was 0.694 ± 0.097 mm in black and 0.712 ± 0.136 mm in white patients with RA; forty-three (35.5%) of the black and 54 (44.3%) of Caucasian patients had plaque. Plaque prevalence and carotid intima-media thickness (cIMT) did not differ between black and white patients in univariate and adjusted analysis.

### 5.2. Major Conventional Risk Factors and RA Characteristics Associated with Atherosclerosis

Upon using interaction terms, population grouping consistently influenced the relations of cardiovascular risk factors with cIMT and plaque. Therefore, cardiovascular risk factor atherosclerosis was determined in stratified analysis, that is, in black and white patients separately. This revealed that systolic blood pressure, the cholesterol-HDL cholesterol ratio, CRP concentrations, and the presence of extra-articular manifestations are independently related to cIMT or/and plaque in white but not black patients with RA. In sharp contrast, the Arthritis Impact Measurement Scales tension score and the use of nonsteroidal anti-inflammatory agents were associated with atherosclerosis in black but not white participants. The Framingham score was significantly associated with atherosclerosis in white but not black patients.

These findings indicate that the atherosclerosis burden is currently as large in black Africans with RA from a developing population as it is in whites from a developed population and further reinforce the notion that adequate cardiovascular risk assessment and management are required in Africans with RA irrespective of ethnicity. Equally important, the findings in this study suggest that major conventional risk factor and systemic inflammation markers are unreliable in cardiovascular risk stratification amongst black Africans with RA. We therefore believe that the systematic use of alternative risk evaluation tools such as vascular imaging by carotid ultrasound [22] may be particularly warranted in this context.

### 5.3. The Relation of Adiposity with Atherosclerosis in African Black Compared to White Women with RA

As obesity is particularly prevalent amongst African Black women, and the potential impact of adiposity on atherosclerosis was examined. Included patients with RA comprised 108 black and 95 white women [53].

BMI and waist-to-height ratio were substantially larger in African black compared to white women with RA (29.9 (6.6) versus 25.3 (4.9) kg/m (*P* = 0.002) and 0.59 (0.09) versus 0.53 (0.08) (*P* = 0.01), resp.).

Anthropometric measures independently associated with the metabolic risk factors of blood pressure, lipid variables, and glucose; population grouping did not impact these relationships. However, in white women, body mass index (BMI) was related to cIMT and adverse fat distribution as estimated by waist-hip ratio associated with plaque; by contrast, none of the anthropometric measures were related to atherosclerosis in African black women with RA. The adiposity-atherosclerosis relations were explained by metabolic risk factors amongst white women with RA.

These findings indicate that obesity as estimated by anthropometric measures in women with RA from developing groups of black African descent does not yet translate into atheroma and hence does not currently represent enhanced atherosclerosis risk, whereas body mass index and waist-to-hip ratio should be considered in cardiovascular risk assessment amongst white women with RA. This supports the notion that optimal CVD risk stratification is likely to differ amongst black and white African women with RA.

### 5.4. The Association of MetS and Its Components with Atherosclerosis in African Black Compared to White Women with RA

The associations between MetS and its components and atherosclerosis were investigated in 104 African black and 93 white women [54].

The MetS and MetS HDL-cholesterol component prevalence were markedly larger in black compared to white female participants (30.8% versus 9.7%; OR (95% CI) = 10.11 (1.76–58.03) (*P* = 0.009) and 21.2% versus 15.1%; OR (95% CI) = 6.14 (1.11–33.92) (*P* = 0.036)), MetS triglycerides and the number of MetS criteria associated independently with plaque in white but not black women with RA. These findings indicate that the current markedly adverse metabolic risk factor profiles in black African patients with RA do not yet represent an enhanced atherosclerosis burden.

## 6. The Potential Impact of Cardiovascular Risk Factors on Early Endothelial Activation in African Black and White RA Patients

In an attempt to further elucidate disparities in CVD risk and its potential effective stratification amongst African black and white RA patients, independent relations of major conventional risk factors and systemic inflammation with surrogate markers of early atherogenesis were examined. The risk factors that were associated with endothelial activation in African black and white patients with RA are also shown in Figure 1. Participants included 112 African black and 105 white patients with RA [55]. Evaluated endothelial activation molecule concentrations included those of E-selectin, vascular adhesion molecule-1, intercellular adhesion molecule-1, and monocyte chemoattractant protein-1. These molecules mediate the initial stages of atherosclerosis and their circulating concentrations associate with prevalent and incident atherosclerosis in RA [20, 21, 56].

In all patients, 3 conventional (smoking, abdominal obesity, and hypertension) and 3 nonconventional cardiovascular risk factors (joint damage, IL-6 concentrations, and prednisone use) were associated with endothelial activation. Interleukin-6 was the only risk factor that was related to each endothelial activation molecule and independently contributed by 18% and significantly more than other risk factors to the variation in overall endothelial activation as estimated by an SD (*z*) score of endothelial activation molecule concentrations. The independent interleukin-6-overall endothelial activation relationships were reproduced in various subgroups. In addition, LDL cholesterol concentrations and the erythrocyte concentrations were associated with endothelial activation in African black but not white patients. Also, disease duration and glomerular filtration rate related to surrogate markers of early atherogenesis in African white but not black participants.

Upon using cardiovascular risk biomarkers, and in contrast to the previously discussed investigations, this study revealed the similarities in CVD risk factor-endothelial activation relations in African black compared to white RA patients mostly. Nevertheless, this investigation again documented that disparities in the potential role of CVD risk factors with possible if not likely implications in cardiovascular risk stratification, exist amongst African black and white patients with RA. Overall, interleukin-6 concentrations are related consistently, markedly, and to a larger extent than other cardiovascular risk factors to endothelial activation in RA. In fact, IL-6 concentrations were numerically more strongly associated with endothelial activation in African black compared to white RA patients partial (*R* = 0.416 versus 0.378). Notably in the present context, IL-6 concentrations were also shown to be associated with coronary artery calcification scores that represent atherosclerosis, in RA [57]. Taken together, the findings support observations alluded to the above, which indicate that there is a need for alternative cardiovascular stratification tools in this case comprising the use of biomarkers, in African black Africans with RA.

## 7. The Relation of Circulating Adipokine Concentrations with Endothelial Activation and Atherosclerosis in Africans with RA

Whereas anthropometric measures, which represent indicators of fat mass, did not relate to atherosclerosis in African black patients with RA, it is now well established that adipose tissue constitutes a highly active endocrine and metabolic organ. Indeed, adipocytes produce a large range of molecules that are referred to as adipocytokines, which mediate the impact of adipose tissue on the risk for CVD and diabetes as well as different bodily functions including immunity, appetite, and energy expenditure [58–64]. Examples of adipokine effects comprise the modulating influence of adiponectin, visfatin, nesfatin, vaspin, and chemerin on obesity-related vascular complications [58, 59] as well as those of adiponectin, leptin, resistin, visfatin, and chemerin on inflammatory and destructive processes [60–63] and cardiovascular risk [64] in RA.

Importantly, in the present context, the production and effects of adipokines can be altered by the presence of autoimmunity [64] and depend on pathophysiological context both in non-RA [59] and RA subjects [64]. Adipokines participate in the pathophysiology of RA and circulating concentrations of leptin [65] and adiponectin [66] relate to metabolic risk whereas those of resistin are associated with systemic inflammation in this disease [67]. Interestingly, visfatin is not associated with inflammation or metabolic syndrome in patients with severe RA [68]. Indeed, the role of adipokines in cardiovascular risk amongst patients with RA remains uncertain. As excess adiposity is highly prevalent in African black patients with RA and adipokines reflect not only fat mass but also adipocyte bioactivity, could the evaluation of adipokine concentrations assist in the exploration of CVD risk and its stratification in this context?

In this regard, several investigations on the relations of adipokines with CVD risk amongst approximately 120 African black and 120 white patients with RA were recently reported. Indeed, adiponectin [69, 70], leptin [71], chemerin [72], and retinol binding protein-4 [73] were associated with metabolic risk factors. More importantly, adiponectin [70, 74], leptin [71, 75], chemerin [72], retinol binding protein 4 [73], and resistin [76, 77] were each independently related to surrogate markers of endothelial activation and atherosclerosis in RA (Figure 1). A detailed account of the different findings is beyond the scope of this review. Pertinently however, the independent relations of each of the 5 studied adipokines with endothelial activation and atherosclerosis were mostly documented in groups stratified on the basis of the presence or absence of different conventional or nonconventional risk factors [70–77]. These findings amply document that pathophysiological context impacts adipokine-CVD risk relations and indeed suggest that adipokine concentrations can contribute to improved CVD stratification in RA.

With regard to population origin, whereas adiponectin and leptin production is increased or unaltered in RA patients from developed populations, circulating concentrations of both adipokines were reduced in black African RA compared to non-RA subjects [69]. However, black population origin did not impact adipokine-endothelial activation [70] and atherosclerosis [74] with a single exception. The latter comprised a paradoxically direct association between adiponectin concentrations and endothelial activation amongst white but not black Africans with RA [70]. In contrast to the other investigated adipokines, adiponectin reduces atherosclerosis risk in non-RA subjects. Paradoxical adipokine-endothelial activation relations in RA likely represent compensatory changes in adipokine production in the presence of increased cardiovascular risk and in an attempt to reduce this risk [70]. Indeed, the paradoxical adiponectin-endothelial activation relation concurred with a borderline significant inverse association of adiponectin with carotid plaque, an indicator of severe, advanced, and high risk atherosclerosis in white RA patients [74].

Adiponectin is further a potential therapeutic target in RA [69]. If our findings are confirmed in future longitudinal and mechanistic studies, then adiponectin inhibition would be expected to potentially enhance CVD risk particularly in white patients with RA.

Overall, these studies indicate that, in contrast to conventional and previously investigated nonconventional cardiovascular risk factors, adipokine concentrations may represent promising tools in CVD risk stratification in black Africans with RA.

## 8. Limitations and Future Perspectives

The most important limitation of currently available data on CVD and its risk factors in African black patients with RA is that they consistently derive from cross-sectionally designed investigations. Longitudinal studies with the additional inclusion of CVD events as an outcome measure in this patient population are underway. Also, formal evaluation of aortic and left ventricular function is needed, particularly considering the high prevalence of hypertension in African black persons including those with RA and heart failure in the African black population at large. Investigations addressing this limitation of previous reports have also been initiated.

Antirheumatic drugs comprising nonsteroidal anti-inflammatory agents (NSAID), corticosteroids, and synthetic and biologic disease modifying agents for rheumatic disease (DMARD) can influence CVD risk in RA [2, 17, 18, 21]. In this regard, investigations performed in the USA documented that markers of sociodemographic disadvantage including black ethnicity associate with less frequent use and later initiation of synthetic and biologic DMARD as well as more regular NSAID use [78–80]. However, with the exception of absent versus infrequent use of biologic DMARD in African black compared to white RA patients, both groups employed similar antirheumatic drug regimens in our settings [51, 53].

## 9. Conclusion

The present review argues not only against the extrapolation of findings on atherogenesis and recommendations on CVD risk stratification derived in non-RA to RA populations but also against that of data originating in patients with RA that belong to developed populations to those from developing populations. In this regard, we found that routinely assessed traditional and nontraditional CVD risk factors were consistently and independently related to atherosclerosis in African white but not black patients with RA. By contrast, circulating concentrations of novel CVD risk biomarkers including interleukin-6 and interleukin-5 adipokines were mostly similarly associated with both endothelial activation and atherosclerosis amongst African black and white RA patients. Reliable CVD risk stratification in African black RA patients may prove to require systematic vascular imaging such as carotid ultrasonography and possibly the use of novel CVD risk markers.

## Figures and Tables

**Table 1:** The epidemiological health transition stages and their characteristics.

Stage	Circumstance	Environmental factors and related CVD risk factors	CVD events
1	Living in rural area	Infections and nutritional deficiencies	Rheumatic heart disease and cardiomyopathy
2	Early urbanization	Reduced infection and improved nutrition, psychosocial stress, and hypertension	Heart failure and hemorrhagic stroke
3	More advanced urbanization	Lifestyle changes: increased fat intake, cigarette smoking, and inactivity	Atherosclerotic CVD and ischemic stroke at young age
4	Established urbanization	Improved health care and CVD prevention	CVD and stroke in the elderly

CVD: cardiovascular disease.

## Abbreviations

RA: Rheumatoid arthritis
AIMS: Arthritis impact measurement scales
NSAID: Nonsteroidal anti-inflammatory agents
LDL: Low density lipoprotein
ESR: Erythrocyte sedimentation rate
C: Cholesterol
HDL: High density lipoprotein
CRP: C-reactive protein
BMI: Body mass index
MetS: Metabolic syndrome
eGFR: Estimated glomerular filtration rate.
